# Supplementary material for: Transcriptome Changes Reveal the Molecular Mechanisms of Humic Acid-Induced Salt Stress Tolerance in Arabidopsis
Source: Molecules. 2021 Feb 3;26(4):782. doi: 10.3390/molecules26040782 (PMC7913487; doi:10.3390/molecules26040782)
Supplement: Supplementary file 1 [file molecules-26-00782-s001.zip › molecules-1079192-sup-org.docx]

Supplementary Materials

 Transcriptome Changes Reveal the Molecular Mechanisms of Humic Acid-Induced Salt Stress Tolerance in Arabidopsis

Joon-Yung Cha ^1,^*, Sang-Ho Kang ^2^, Myung Geun Ji ^1^, Gyeong-Im Shin ^1^, Song Yi Jeong ^1^, Gyeongik Ahn ^3^, Min Gab Kim ^4^, Jong-Rok Jeon ^3^ and Woe-Yeon Kim ^1,3,^*

^1^ Division of Applied Life Science (BK21four), Plant Molecular Biology and Biotechnology Research Center, Research Institute of Life Sciences, Gyeongsang National University, Jinju 52828, Republic of Korea; jycha@gnu.ac.kr (J.Y.C.); mgj2930@gnu.ac.kr (M.G.J.); shin123@gnu.ac.kr (G.I.S.); songyi123@gnu.ac.kr (S.Y.J.); kim1312@gnu.ac.kr (W.Y.K.)

^2^ Genomics Division, National Institute of Agricultural Sciences, Rural Development Administration, Jeonju 54874, Republic of Korea; hosang93@korea.kr (S.H.K.)

^3^ Department of Agricultural Chemistry and Food Science & Technology, Institute of Agriculture and Life Science, Gyeongsang National University, Jinju 52828, Republic of Korea; ahngi@gnu.ac.kr (G.A.); jrjeon@gnu.ac.kr (J.R.J.); kim1312@gnu.ac.kr (W.Y.K.)

^4^ College of Pharmacy and Research Institute of Pharmaceutical Science, Gyeongsang National University, Jinju 52828, Republic of Korea; mgk1284@gnu.ac.kr (M.G.K)

* Correspondence: jycha@gnu.ac.kr (J.Y.C.) & kim1312@gnu.ac.kr (W.Y.K.); Tel.: +82-55-772-1968 (J.Y.C. & W.Y.K.); Fax: +82-55-772-2631 ((J.Y.C. & W.Y.K)

**Table 1.** Mapping statistics for quality filtered reads generated for Arabidopsis seedlings.

| **Treatments** | **Total reads and**  **percentage** | **Raw** | **Clean** | **Mapped** | **Uniquely**  **Mapped** | **Unmapped** |
| --- | --- | --- | --- | --- | --- | --- |
| Salt-Set1 | Reads | 61664692 | 61116414 | 58676027 | 57715479 | 2440387 |
|  | Percenage (%) | 100 | 0.99 | 0.96 | 0.94 | 0.04 |
| Salt-Set2 | Reads | 53463110 | 52812694 | 50260307 | 49381609 | 2552387 |
|  | Percenage (%) | 100 | 0.99 | 0.95 | 0.94 | 0.05 |
| Salt-Set3 | Reads | 45751666 | 45167010 | 43414749 | 42839470 | 1752261 |
|  | Percenage (%) | 100 | 0.99 | 0.96 | 0.95 | 0.04 |
| Salt + HA-Set1 | Reads | 56060428 | 55395210 | 53083622 | 52193562 | 2311588 |
|  | Percenage (%) | 100 | 0.99 | 0.96 | 0.94 | 0.04 |
| Salt + HA-Set2 | Reads | 43785138 | 43293310 | 41295288 | 40421515 | 1998022 |
|  | Percenage (%) | 100 | 0.99 | 0.95 | 0.93 | 0.05 |
| Salt + HA-Set3 | Reads | 51329056 | 50759750 | 48875042 | 48025852 | 1884708 |
|  | Percenage (%) | 100 | 0.99 | 0.96 | 0.95 | 0.04 |

**Table S2.** Primers used in this study.

| **Gene name** | **Primer name** | **Primer sequence** | **Purpose** |
| --- | --- | --- | --- |
| *ROXY10* | *ROXY10-For* | 5’-GCTGTTGGATCATATACCATATAAAATAGC-3’ | qRT-PCR |
|  | *ROXY10-Rev* | 5’-CGCAACAACGCCTGCTCGATCT-3’ |  |
| *ROXY12* | *ROXY12-For* | 5’-GTGAACAAAGAAAACATACACAAGAATGTATG-3’ |  |
|  | *ROXY12-Rev* | 5’-AGAACCAGTGTTTACAAAATAGGCAACCGGA-3’ |  |
| *ROXY13* | *ROXY13-For* | 5’-ACTCTAAGTCATCTCTTATATATCGTCAGCCA-3’ |  |
|  | *ROXY13-Rev* | 5’-GGTATTATAATGTGAAATGTGTTCGAAGAGCGA-3’ |  |
| *HSP101* | *HSP101-For* | 5’-TGGTCATCGGCTATTTCTCC-3’ |  |
|  | *HSP101-Rev* | 5’-GCTTGTTGTCGTTGCTTTCC-3’ |  |
| *HSP81.1* | *HSP81.1-For* | 5’-CCACTGATGTAGCAGCAAGG-3’ |  |
|  | *HSP81.1-Rev* | 5’-CAGAACCAGAGGATCGGACT-3’ |  |
| *TT5* | *TT5-For* | 5’-CATCGATCCTCTTCGCTCTC-3’ |  |
|  | *TT5-Rev* | 5’-AGGTGACACACCGTTCTTCC-3’ |  |
| *TUB* | *TUB-For* | 5’-TGGCATCAACTTTCATTGGA-3’ |  |
|  | *TUB-Rev* | 5’-ATGTTGCTCTCCGCTTCTGT-3’ |  |

|  | **Group 1** | **Group 2** | **Sum** | **Up** | **Down** |
| --- | --- | --- | --- | --- | --- |
| **DEG #1** | MS | Salt | 3,785 | 1,625 | 2,160 |
| **DEG #2** | MS | HA | 3,257 | 1,677 | 1,580 |
| **DEG #3** | Salt | Salt + HA | 5,271 | 2,483 | 2,788 |
| **DEG #4** | HA | Salt + HA | 2,582 | 1,075 | 1,507 |

**a**

**b**

-

500

1,000

1,500

2,000

2,500

3,000

DEG #1

DEG #2

DEG #3

DEG #4

Up

Down

Number of DEGs

**Supplementary Figure S1.** Number of expressed genes by treatment. (**a**) Number of expressed genes in each treatment averaged for three replicates and DEG sets for comparison. (**b**) Number of differentially (up- or down-) regulated genes in each DEG set.


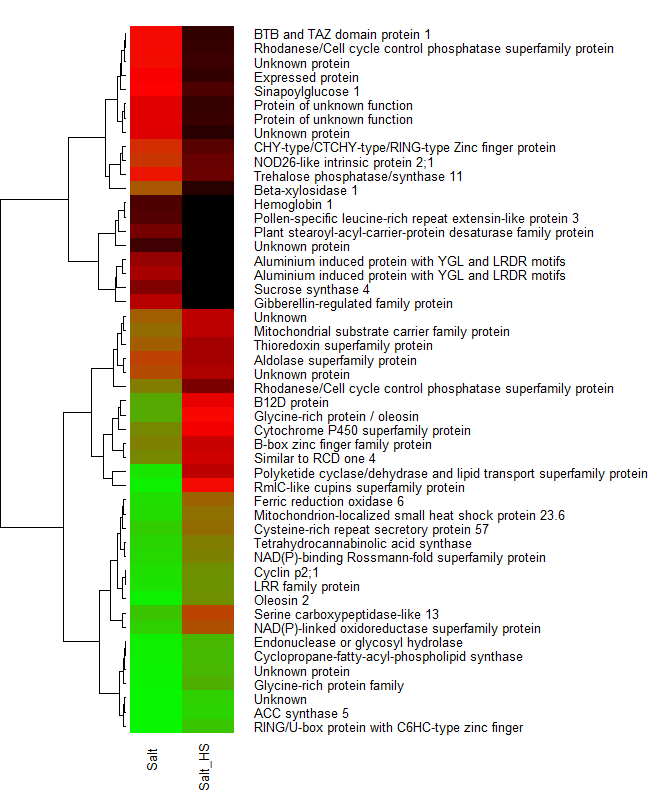

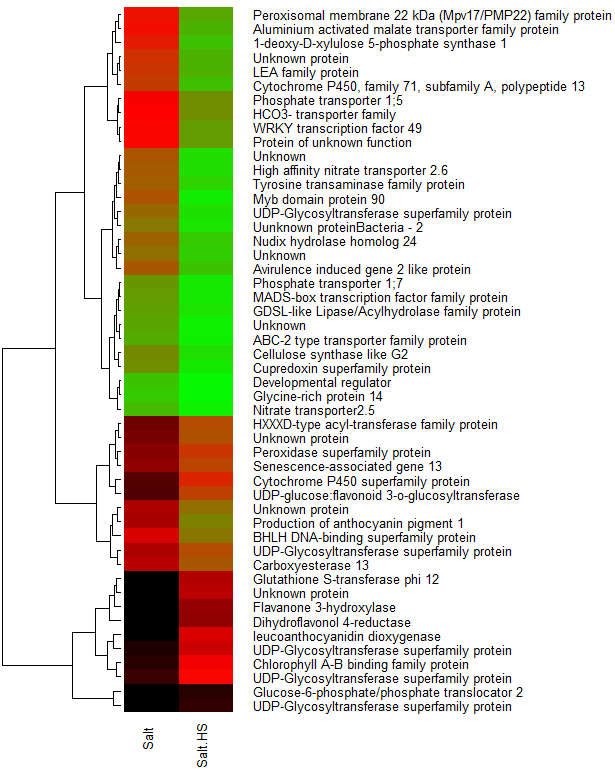

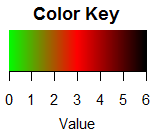


**a**

**b**

Salt

Salt+HA

Salt

Salt+HA

**Supplementary Figure S2.** Transcript levels of the most differentially regulated genes in DEG #3. Transcript levels of the 50 most up-regulated (**a**, log_2_ fold change ≥ 1) or down-regulated (**b**, log_2_ fold change ≤ -1) genes in DEG #3 represented as a heatmap.


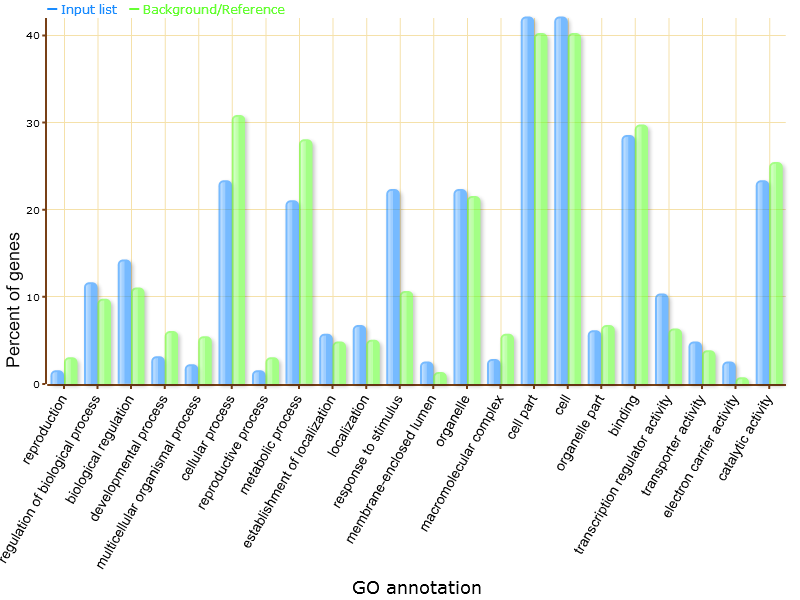


Biological process

Cellular component

Molecular function

**Supplementary Figure S3.** GO term enrichment analysis of up-regulated genes in the main categories biological process, cellular component, and molecular function in DEG #3. The percentage of genes in our input list was compared to that in the background/reference.


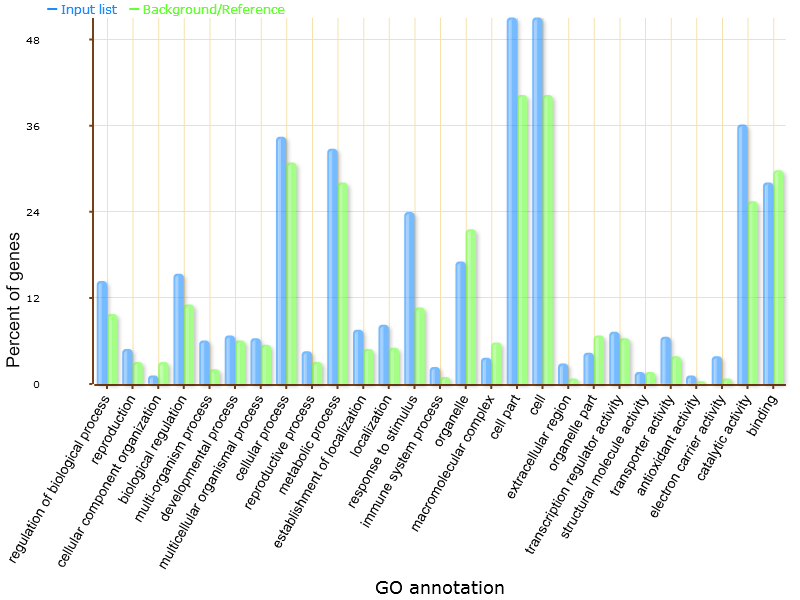


Biological process

Cellular component

Molecular function

**Supplementary Figure S4.** GO term enrichment analysis of down-regulated genes in the main categories biological process, cellular component, and molecular function in DEG #3. The percentage of genes in our input list was compared to that in the background/reference.


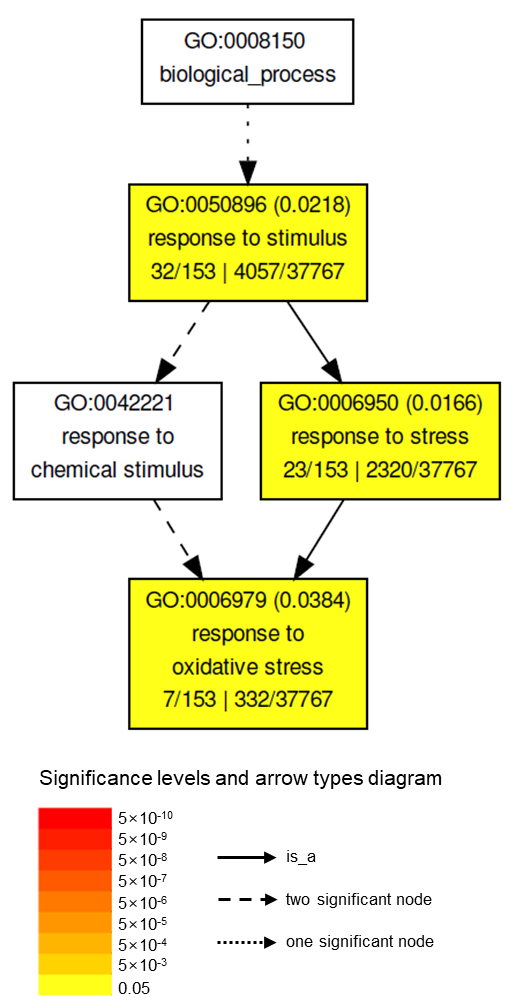


**Supplementary Figure S5.** GO term enrichment analysis of genes up-regulated in both DEG #2 and DEG #3. Representations are described in the Figure 3b legend.


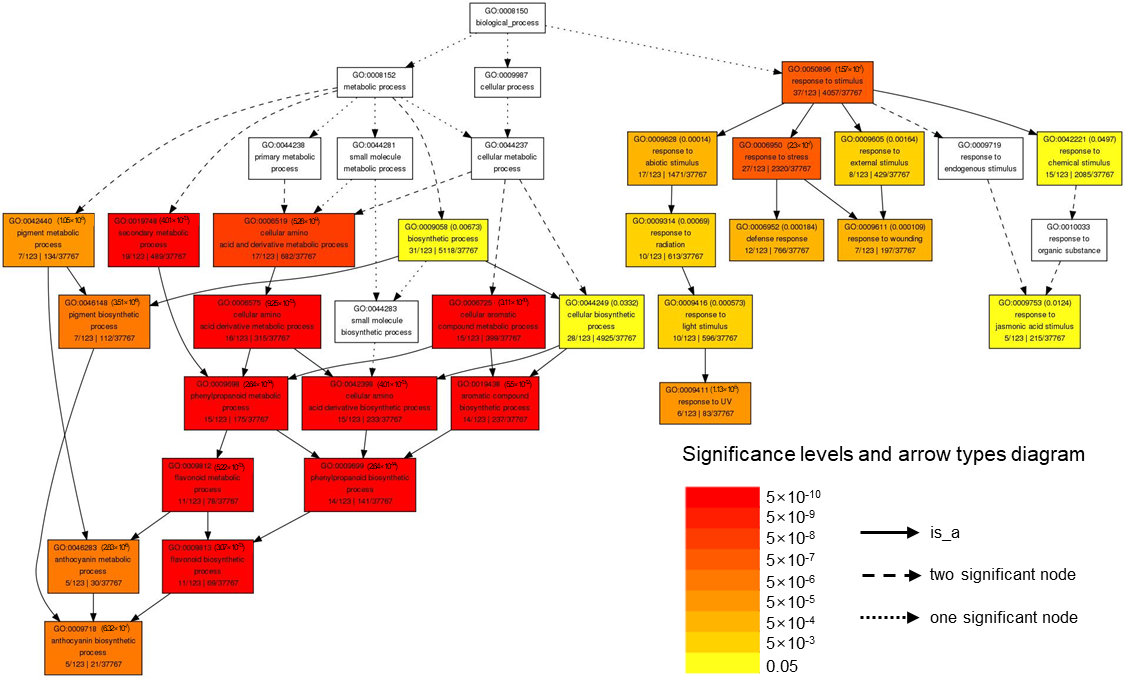


**Supplementary Figure S6.** GO term enrichment analysis of genes down-regulated in both DEG #2 and DEG #3. Representations are described in the Figure 3b legend.
